# Sense of Coherence and Health-Related Quality of Life in Patients with Multiple Sclerosis: The Role of Physical and Neurological Disability

**DOI:** 10.3390/jcm11061716

**Published:** 2022-03-19

**Authors:** Joanna Dymecka, Rafał Gerymski, Rafał Tataruch, Mariola Bidzan

**Affiliations:** 1 Department of Health Psychology and Quality of Life, Institute of Psychology, Opole University, 45-040 Opole, Poland; jdymecka@uni.opole.pl; 2 Faculty of Physical Education and Physiotherapy, Opole University of Technology, 45-758 Opole, Poland; r.tataruch@po.edu.pl; 3 Department of Clinical and Health Psychology, Institute of Psychology, University of Gdansk, 80-309 Gdansk, Poland; mariola.bidzan@ug.edu.pl

**Keywords:** multiple sclerosis, physical disability, neurological disability, sense of coherence, quality of life

## Abstract

Multiple sclerosis is a progressive demyelinating disease of the central nervous system that can lead to permanent disability and significantly impact the quality of life. The present study explores the relationship between neurological disability and disease symptoms, quality of life, and the level of sense of coherence, which is an important resource for coping with the disease. EDSS, GNDS, SOC-29, and MSIS-29 were used in the presented study. The study group consisted of 137 people diagnosed with multiple sclerosis. The study showed the correlation between most of the analyzed variables. Mood disability and fatigue were significant predictors of the sense of coherence and explained 34% of its variance. Physical disability and fatigue served as significant predictors of the physical aspect of quality of life, explaining 49% of its variance. Fatigue and sense of coherence were significant predictors of the psychological aspect of quality of life, explaining 47% of the variance. In studies on a group of people with multiple sclerosis, it is worth considering, in addition to the level of disability, also invisible symptoms, such as fatigue or mood disability, which are important for the quality of life and the level of resources. As this study suggests, a sense of coherence is a resource essential for the quality of life of people with disabilities.

## 1. Introduction

Multiple sclerosis (MS) is a chronic, progressive demyelinating disease with a not fully understood etiology. MS is the most common cause of neurological disability in young adults. This disease is the result of damage to the myelin sheath in the central nervous system. The course of MS is unpredictable, and the disease is characterized by various clinical courses. During its duration, many neurological symptoms appear, such as difficulties in movement, paresis of the upper and lower limbs, sensory and visual disturbances, speech problems, bladder and bowel dysfunction, chronic pain, fatigue, and cognitive and emotional problems, which may significantly affect the functioning of the patient [1,2,3].

Multiple sclerosis is a disease that has no direct effect on life expectancy, and therefore, patients struggle with it for many years. It begins in early adulthood, affects professional activity and family planning, and since it is unpredictable and is associated with a wide range of neurological symptoms, including cognitive impairment and depression [4,5,6], it constitutes an extraordinary challenge for the psychological functioning of an individual and significantly influences the health-related quality of life (HRQoL). The HRQoL of people with MS is lower compared with healthy people [7,8,9,10,11,12] and people with other chronic diseases such as enteritis, rheumatoid arthritis, epilepsy, diabetes, and cardiovascular diseases [13,14]. The quality of life of people with MS is influenced, among other factors, by the degree of disability, neurological symptoms such as problems in the functioning of the lower and upper limbs, fatigue, depression, and cognitive disorders [14,15]. It has also been shown that many psychosocial factors [16,17,18,19,20,21,22,23,24,25] also affect the quality of life in people with MS. Some researchers emphasize that resources are often more important than biological issues [14] because the influence of many negative factors may be weakened by the functioning of protective factors [14], which undoubtedly include the sense of coherence (SOC), defined as “a global orientation that expresses the extent to which one has a pervasive, enduring though dynamic feeling of confidence that (1) the stimuli deriving from one’s internal and external environments in the course of living are structured, predictable, and explicable; (2) the resources are available to one to meet the demands posed by these stimuli; and (3) these demands are challenges worthy of investment and engagement” [26].

SOC seems to be a particularly important variable in the process of coping with multiple sclerosis. The high level of SOC makes it easier to accept the inevitable difficulties [27]. High SOC is especially necessary for an individual who is affected by strong stressors [28]. Multiple sclerosis is a disease that threatens human health and efficiency. MS is unpredictable and cannot be prevented, and it is also associated with a very strong stress, so a high sense of coherence may turn out to be a particularly important variable that protects an individual through years of struggling with a disease such as MS.

The theoretical assumptions of Antonovsky’s concept [26] and the results of many studies provide the basis for research on a relationship between variables related to the course of MS, such as the level of disability and the severity of disease symptoms, the sense of coherence, and health-related quality of life. The level of disability in MS may affect a patient’s quality of life. Moreover, numerous studies have shown that people with chronic diseases have a significantly lower sense of coherence than healthy people [29,30,31,32] and that the level of resources differs depending on the type of disease [33], which may indicate that the variables related to the course of a disease—such as, e.g., disability and symptoms—may affect the level of sense of coherence. On the other hand, SOC itself is a resource that protects a person against the negative impact of life situations [28].

The role of SOC as a significant predictor of quality of life has been demonstrated in many previous studies and analyses that proved that the higher the SOC, the better the quality of life [34,35,36]. The importance of SOC for life satisfaction has also been demonstrated during the COVID-19 pandemic [37]. Studies have shown that people with high levels of coherence can adapt to stress despite adversity and can maintain good health and quality of life [38]. Therefore, this study aimed to analyze the relationship between disability, disease symptoms, the sense of coherence, and health-related quality of life.

## 2. Method

### 2.1. Participants and Procedure

The study group consisted of 137 people diagnosed with multiple sclerosis—73 women and 64 men aged between 18 and 73 (*M* = 46.47; *SD* = 12.59). They were patients staying at rehabilitation camps at the Rehabilitation Centre for People with Multiple Sclerosis in Borne Sulinowo (Poland) and the members of the foundations and associations providing help to people with MS—the Association of Patients with Multiple Sclerosis in Głogów (Poland) and the “Twardziele” group (Tricity, Poland). Patients with cognitive deficits hindering the understanding of psychological questionnaires—i.e., patients who obtained more than 3 points in the cognitive disorders subscale in the GNDS questionnaire—were excluded from the study. The mean duration of MS in the study sample was 14.61 years (*SD* = 8.31). Disease-modifying therapy was used by 62.04% of the studied patients, and 37.96% had never had access to this type of therapy. Only about 8% of patients participated in drug programs for more than 5 years. For more detailed information, see Table 1.

The examination was carried out during one meeting with the patient. The meeting had no time limit, and its duration was adjusted to the psychophysical abilities of the patient. The full psychological examination lasted from about 45 min to 2 h. Before participating in the study, the patients were asked to give their full consent. They were informed about the purpose of the research, its anonymity, and that all data would be used for research purposes only. The study consisted in completing a set of questionnaires by patients, which were always given in the same order. The study was approved by the Ethics Committee at the Institute of Psychology of the University of Gdańsk (No. 19/06/2015). All respondents gave their consent to participate in the study.

### 2.2. Measures

Physical disability was operationalized with the Expanded Disability Status Scale (EDSS) [3,39]. It is widely used to evaluate the degree of neurologic impairment among MS patients. The EDSS includes 20 degrees of disability, scored every half grade. The higher the score on the scale, the greater the disability, where: 1—no disability; 5—disability severe enough to impair full daily activities; 10—death due to MS. Due to EDSS’s nature, standard reliability coefficients such as Cronbach’s *alpha* cannot be calculated for it.

Neurological disability was measured with the Guy’s Neurological Disability Scale (GNDS) [40,41]. It consists of 12 subscales concerning individual areas of functioning, measured with 72 questions on a dichotomous nominal scale (where 1—*Yes*; 0—*No*): cognitive disorders, mood disability, problems with eyesight, speech, swallowing, upper limb functioning, lower limb function, functioning of the bladder and intestines, problems with sexual functioning, and fatigue. A high score on a particular scale indicates a high level of the perceived symptoms. GNDS shows good reliability (in the presented study, Cronbach’s *alpha* = 0.73).

The Sense of Coherence Scale (SOC-29) [42,43] was also used in this study. The questionnaire consists of 29 items related to various aspects of human life. The study participants responded to them on a seven-point scale. The questionnaire is used to study the global sense of coherence and its three components: the senses of comprehensibility, manageability, and meaningfulness. Only the global score was used in this study. The scale shows good reliability (in this study Cronbach’s α was 0.91).

The physical and psychological impact of MS was measured by the Multiple Sclerosis Impact Scale 29 (MSIS-29) [44,45]. The scale consists of 29 questions: 20 regarding an individual’s physical condition and 9 regarding their psychological condition. Participants assess each of the items on a 5-point Likert scale, ranging from 1 (*Not at all*) to 5 (*Extremely*). The higher the score, the higher the impact of MS on one’s sphere of functioning. The reliability of the Polish version of the scale is acceptable (in the presented study, Cronbach’s *alpha* = 0.83–0.87).

### 2.3. Data Analysis

The *t*-test analysis was used to verify the significance of the differences between studied groups. Pearson’s r correlation and backward stepwise regression were used to estimate the relationships between selected variables. Pearson’s r correlation was used even though one of the tested variables was represented on an ordinal scale—the results of the EDSS. In the case of this type of scale, non-parametric analyses are usually used. However, parametric tests show much higher statistical power [46,47]. Many simulations have shown that parametric analyses are resistant to breaking their basic assumptions, such as the normality of the distribution of the tested variables, variance homogeneity, or the type of measuring scale and that they can also be used for ordinal scales due to their robustness [48,49,50,51]. Therefore, in the results section of this article, only parametric analyzes were used due to their statistical power and low asymmetry of the tested distributions. What is more, a backward stepwise regression approach was used due to a large number of variables, even though no multicollinearity was detected (all VIF coefficients were below 10). Analyses were conducted using the IBM SPSS 24. All statistical tests were two-tailed, and the significance level was set to α = 0.05.

## 3. Results

### 3.1. Descriptive Statistics and the Test Selection

First, the descriptive statistics of the studied variables were analyzed. These data are presented in Table 2. The results of the Shapiro–Wilk test showed that the distributions of most of the examined variables were non-normal. However, the analysis of the values of skewness and kurtosis coefficients shows that the distributions of 16 out of 19 examined variables were not characterized by large asymmetry—the exceptions were visual disability, speech disability, and swallowing disability scores [52].

### 3.2. Group Homogeneity

The first step of the statistical analysis verified whether the studied group of patients was a homogeneous sample. Gender comparisons were calculated using the *t*-test analysis. The analysis did not show any significant gender differences in any of the tested variables: physical disability, neurological disability, and its symptoms, sense of coherence, or the physical and psychological impact of MS. For more detailed information, see Table 3. Based on the presented results, it was decided to treat the studied sample as homogeneous in further analyses.

### 3.3. Relationship between Studied Variables

Pearson’s *r* correlation was used to verify the relationships between the studied variables. First, only the total scores of the administered scales were used. Analysis showed that the physical disability score was significantly and positively related to neurological disability (moderate strength), the physical impact of the MS (strong strength), and the psychological impact of the MS (weak strength) scores. The relationship between physical disability and sense of coherence was non-significant. What is more, the neurological disability score was negatively related to the sense of coherence (moderate strength) and positively related to the physical (strong strength) and psychological impact (moderate strength) of the MS scores. Additionally, the sense of coherence was negatively related to the physical (weak strength) and psychological impact (strong strength) of the MS scores. For more detailed information, see Table 4.

It was also decided to verify which symptoms of neurological disability were related to the results of the sense of coherence and the physical and psychological impact of MS. Comprehensibility and manageability scales were significantly and negatively related to cognitive disability (weak strength), mood disorder (moderate strength), and fatigue (moderate strength). The meaningfulness scale was significantly and negatively related only to the mood disorder (moderate strength) scores. The physical impact of the MS score was significantly and positively related to the mood disability (weak strength), upper limb, lower limb, bladder, intestines and sexual disabilities, and fatigue (moderate strengths) scores. The psychological impact of the MS was related to cognitive disability, mood disorder (moderate strength), speech and bowel disability (weak strength), and fatigue (moderate strength). For more detailed information, see Table 5.

In the last step, it was decided to verify which of the studied variables would be statistically significant predictors of the sense of coherence and the impact of MS on the physical and psychological spheres. For this purpose, three backward stepwise regression analyses were used. The selection of the predictors was made based on the results obtained in the Pearson’s *r* correlation. We declared predictors in the model based on the variables that were significantly related to the selected dependent variable. Mood disability and fatigue were significant predictors of the sense of coherence and explained 34% of its variance. Additionally, physical disability and fatigue served as significant predictors of the physical impact of the MS scores, explaining 49% of the dependent variable’s variance. What is more, fatigue and sense of coherence were significant predictors of the psychological impact of the MS scores, explaining 47% of the variance. For more detailed information, see Table 6.

## 4. Discussion

The study aimed to determine the relationship between disability and symptoms of multiple sclerosis and the sense of coherence and health-related quality of life. The study showed a correlation between most of the analyzed variables. Motor impairment (EDSS), neurological disability (GNDS), and most of the symptoms were related to the level of health-related quality of life. Disability and fatigue were significant predictors of the physical aspect of HRQoL. On the other hand, the predictors of the psychological aspect of HRQoL were fatigue and a sense of coherence.

The level of the sense of coherence was not related to physical disability measured by the EDSS. This may be related to the characteristics of the EDSS scale, a scale that is focused primarily on one functional area and that emphasizes walking abilities [3]. On the other hand, SOC was related to neurological disability measured with GNDS, which also includes psychological problems such as mood disability, cognitive impairment, and fatigue, which may, more than the physical disability itself, affect the level of resources such as the sense of coherence. Disability and its rapid progress are associated with a high level of stress and negative emotions, which also affects the level of the sense of coherence. Mood disability as a symptom of MS, which was measured by the GNDS scale, can also lower SOC levels. Although SOC appears to be a stable variable [26], selected MS symptoms may affect its level, which was confirmed in this study.

Among the symptoms of MS, the sense of coherence was the construct most strongly associated with mood disability. Mood disability was also the most important predictor of the level of SOC in the presented study. Studies show that depression is one of the more common symptoms of MS and that in this group of patients it occurs more often than in other people with chronic diseases and much more often than in the general population [53,54,55,56]. Mood disability can affect the perception of oneself and the world, self-esteem, activity, and motivation. The relationship between the sense of coherence and mood disability has been confirmed in many studies among various groups of patients. Depression is associated with a low level of sense of coherence [57,58]. A negative correlation between depression and SOC has been shown in patients with various chronic diseases, including ischemic heart disease and type 1 diabetes [33], in patients after cardiological surgery [59], as well as in oncological [32,59]. There are also reports that over time the sense of coherence has less impact on the symptoms of depression, while depression increasingly affects the sense of coherence [60].

Another symptom of multiple sclerosis—fatigue—is defined as a feeling of exhaustion with a need to rest or a subjective feeling of lack of physical or mental energy that interferes with daily activities. It is described by 40–50% of patients as the most severe symptom of the disease [61,62]—more burdensome than pain or even physical disability [63]. It acted as a significant predictor of the SOC and HRQoL in the presented study. Fatigue may affect the daily functioning and the MS patients’ ability to cope with everyday demands. Fatigue is the main cause of early retirement. It can lead to an increase in existing disability and can adversely affect rehabilitation outcomes [19].

The results of the current study indicate that mood disturbances and fatigue were predictors of the sense of coherence. The sense of coherence is one of the most important variables that can help with effective coping with stress [64], including those related to chronic disease; however, it may itself be influenced by negative life experiences. According to the statement of Antonovsky [26], the level of the sense of coherence is established around the age of 30; however, some researchers who believe that critical life events may cause changes in SOC level also in a later period [26] considered SOC to be a stable life orientation. He associated the stability of the sense of coherence with the fact that each person has a characteristic average SOC level that is appropriate for them; however, he drew attention to the fact that there may be states of temporary fluctuations in the SOC level—e.g., after highly traumatic events. It is believed that particularly difficult events, such as severe somatic disease, can lower the sense of coherence [65]. It was also found in research that negative life events lead to a decrease in the sense of coherence [66]; however, the lack of SOC measurements before the occurrence of negative life events makes it difficult to determine whether that situation led to a decrease in the SOC level [67]. The relationship between the sense of coherence and neurological disability and some symptoms, proven in the presented study, suggests that it is the disability, mood disability, and fatigue that may affect the SOC level in people with multiple sclerosis. Other studies have also found that a reduced SOC can persist for up to two years after a highly stressful event, such as the occurrence of a diagnosis of chronic disease [68]. There is an opinion that especially people with a low and medium level of the sense of coherence are exposed to its reduction under the influence of negative life events since people with high SOC levels are more resistant to negative changes [69].

The importance of the sense of coherence for the functioning of people with chronic diseases has been demonstrated in many previous studies. Studies show that SOC is an important protective factor in the first year after cancer diagnosis [70]. It was found that the sense of coherence makes the disease perceived as less threatening [71] and less stressful [68] and can act as a buffer protecting against the harmful effects of perceived stress [72,73]. Multiple sclerosis is considered to be a stressor of particular intensity, inter alia, due to the wide range of symptoms that occur during its duration. Dudek and Koniarek [74] indicate that the effect of SOC is most important in people who are under the influence of strong stress. The authors believe that strong SOC is especially important when the individual experiences very difficult situations, such as having MS.

The impact of the sense of coherence on coping with stressful situations may be related to the fact that it reduces the experience of negative emotions in difficult situations. The research results confirm that SOC may limit the experience of negative emotions in stressful situations [27], which may have a beneficial effect on functioning during the disease. Among people with a physical disability after limb amputation or spine surgery, it was found that patients whose sense of coherence level was comparable with the SOC of healthy people did not despair because of their health condition, but they tried to give a new meaning to their lives [75], which may have a positive effect on health-related quality of life.

The present study has shown that the sense of coherence was related to HRQoL. The sense of coherence was more strongly associated with the mental aspect of quality of life compared with the physical aspect. Another study supports the presented results, showing that SOC was the strongest predictor of the psychological aspect of quality of life, explaining 35.6% of its variance, while it was a less significant predictor of the physical quality of life of multiple sclerosis patients [76]. It has also been shown that in MS patients, higher SOC was associated with better mental health, less negative affect, and less depression [77]. Another study among people with multiple sclerosis showed that patients with fewer limitations in functioning and with a higher level of sense of coherence had a higher quality of life [78]. Additionally, in research by Kossakowska [79] it was shown that one of the most important factors determining the quality of life of people with multiple sclerosis is the sense of coherence, which affects the subjective assessment of physical functioning and life satisfaction. The sense of coherence in Kossakowska’s research was the most important variable associated with a high level of HRQoL in people with MS—definitely more important than self-esteem and optimism. The relationship between the sense of coherence and the quality of life was also demonstrated among other groups of patients, including those with cardiovascular diseases [59,80,81,82] or with cancer [59,71,83].

Unfortunately, like any other research project, this one is not free from limitations. First, our results were based on a cross-sectional study. Longitudinal studies should be conducted to verify the predictive role of the studied variables in the HRQoL of MS patients’. Our paper discusses the obtained results based on the theoretical background, without any definite causal conclusions. Longitudinal research is needed to determine the causality and the predictive value of the tested variables. Second, the sample was treated in the analyses as a homogeneous group. However, this does not mean that all respondents were very similar to each other. The omission of testing for other biomedical and psychological variables is a major limitation of the presented research project and should be taken into account in future studies [19]. Lastly, the presented sample size is also an important limitation of this manuscript. To strengthen the conclusions contained in this paper, a larger sample of people should be tested.

## 5. Conclusions

This study confirms the results obtained in previous studies, both in the case of multiple sclerosis and other chronic diseases, which found that the sense of coherence is important for health-related quality of life. The sense of coherence can protect a person from the negative impact of stress related to the symptoms of the disease and can reduce their negative impact on the quality of life. The sense of coherence is conducive to effective coping with the disease and has a positive impact on the quality of life. People with a high sense of coherence deal with the disease more effectively and have a higher quality of life. At the same time, it should be noted that the level of SOC may be influenced by other variables, especially those related to invisible symptoms of the disease, such as depression and fatigue. Therefore, when working with people with multiple sclerosis, it is important to pay attention not only to the physical functioning of patients or the ability to move but also to symptoms of a psychological nature, such as depression, cognitive impairment, and fatigue. Therefore, a psychiatrist, psychologist, or neuropsychologist should be involved in working with the patient. Only an interdisciplinary approach to multiple sclerosis will improve the quality of life of this group of patients.

## Figures and Tables

**Table 1:** Characteristics of the studied sample.

	*M*	*SD*	*Min*	*Max*
Age	46.47	12.59	18.00	73.00
Age of the diagnosis	33.94	10.65	15.00	61.00
Illness duration (in years)	14.61	8.31	<1.00	42.00
	*n*		%	
Gender				
Women	73		53.28%	
Men	64		46.72%	
Education				
Elementary School	2		1.46%	
Vocational	25		18.25%	
High School	58		42.34%	
University—Bachelor’s Degree	13		9.49%	
University—Master’s Degree	38		27.74%	
No data	1		0.73%	
Form of the Multiple Sclerosis				
Relapsing-remitting	43		31.39%	
Primary progressive	22		16.06%	
Secondary progressive	31		22.63%	
Progressive-relapsing	8		5.84%	
Undefined	33		24.09%	

**Table 2:** Descriptive statistics for the tested variables.

	*M*	*SD*	*Me*	*Min*	*Max*	*SKE*	*K*	*W*	*p*
Physical disability (EDSS)	4.57	2.10	4.50	0.00	9.00	−0.14	−0.69	0.97	0.004
Neurological disability (GNDS)	16.98	8.31	16.00	0.00	37.00	0.33	−0.24	0.98	0.052
Cognitive disability (GNDS)	1.04	1.08	1.00	0.00	3.00	0.59	−0.97	0.82	<0.001
Mood disability (GNDS)	1.47	1.32	1.00	0.00	5.00	0.67	−0.33	0.88	<0.001
Visual disability (GNDS)	0.52	0.74	0.00	0.00	3.00	1.60	2.67	0.68	<0.001
Speech disability (GNDS)	0.49	1.02	0.00	0.00	4.00	2.08	3.20	0.54	<0.001
Swallowing disability (GNDS)	0.36	0.82	0.00	0.00	4.00	2.54	6.43	0.51	<0.001
Upper limb disability (GNDS)	1.36	1.25	1.00	0.00	5.00	0.57	−0.40	0.87	<0.001
Lower limb disability (GNDS)	2.16	1.49	2.00	0.00	5.00	0.44	−0.65	0.91	<0.001
Bladder disability (GNDS)	2.64	1.92	2.00	0.00	5.00	−0.04	−1.47	0.85	<0.001
Bowel disability (GNDS)	1.07	1.23	1.00	0.00	5.00	0.97	0.19	0.81	<0.001
Sexual disabilities (GNDS)	2.08	1.92	2.00	0.00	5.00	0.27	−1.46	0.84	<0.001
Fatigue (GNDS)	2.86	1.73	3.00	0.00	5.00	−0.26	−1.20	0.89	<0.001
Sense of coherence (SOC-29)	130.84	29.61	131.00	71.00	194.00	0.01	−0.82	0.98	0.064
Comprehensibility (SOC-29)	45.66	12.20	46.00	11.00	75.00	−0.13	−0.27	0.99	0.257
Manageability (SOC-29)	46.69	11.15	47.00	19.00	69.00	−0.03	−0.64	0.99	0.223
Meaningfulness (SOC-29)	38.49	9.66	38.00	15.00	55.00	−0.27	-0.85	0.97	0.003
Physical impact of MS (MSIS-29)	51.62	19.33	51.00	20.00	97.00	0.33	−0.64	0.97	0.008
Psychological impact of MS (MSIS-29)	23.74	9.48	24.00	9.00	43.00	0.16	−0.95	0.96	0.001

Note: *SKE*—skewness; *K*—kurtosis; *W*—Shapiro–Wilk’s statistic.

**Table 3:** Results of the *t*-test comparisons.

	Women		Men		*t* (*df*)	*p*	*d_Cohen_*
	*M*	*SD*	*M*	*SD*			
Physical disability (EDSS)	4.29	2.19	4.90	1.97	−1.71 (135)	0.090	0.29
Neurological disability (GNDS)	17.45	8.26	16.44	8.39	0.71 (135)	0.478	0.12
Cognitive disability (GNDS)	1.18	1.08	0.89	1.06	1.57 (135)	0.119	0.27
Mood disability (GNDS)	1.49	1.21	1.45	1.45	0.18 (135)	0.861	0.03
Visual disability (GNDS)	0.53	0.77	0.50	0.71	0.27 (135)	0.788	0.05
Speech disability (GNDS)	0.40	0.88	0.59	1.16	−1.12 (135)	0.263	0.19
Swallowing disability (GNDS)	0.37	0.77	0.36	0.88	0.07 (135)	0.941	0.01
Upper limb disability (GNDS)	1.30	1.28	1.44	1.23	−0.63 (135)	0.528	0.11
Lower limb disability (GNDS)	1.96	1.45	2.39	1.51	−1.71 (135)	0.090	0.29
Bladder disability (GNDS)	2.81	1.97	2.45	1.87	1.08 (135)	0.283	0.19
Bowel disability (GNDS)	1.25	1.30	0.86	1.13	1.85 (135)	0.066	0.32
Sexual disabilities (GNDS)	1.95	2.01	2.20	1.84	−0.60 (84) ^#^	0.551	0.13
Fatigue (GNDS)	3.10	1.73	2.59	1.70	1.72 (135)	0.087	0.30
Sense of coherence (SOC-29)	128.49	28.32	133.52	31.02	−0.99 (135)	0.324	0.17
Comprehensibility (SOC-29)	44.51	11.81	46.97	12.60	−1.18 (135)	0.240	0.20
Manageability (SOC-29)	45.22	10.84	48.36	11.35	−1.66 (135)	0.100	0.28
Meaningfulness (SOC-29)	38.75	9.31	38.19	10.10	0.34 (135)	0.734	0.06
Physical impact of MS (MSIS-29)	51.59	20.02	51.66	18.68	−0.02 (135)	0.984	<0.01
Psychological impact of MS (MSIS-29)	24.60	9.73	22.77	9.16	1.13 (135)	0.259	0.19

Note: ^#^ only 86 patients answered the questions about sexual disabilities.

**Table 4:** Results of the Pearson’s r correlation.

	*M*	*SD*	1.	2.	3.	4.	5.
Physical disability (EDSS)	4.57	2.10	-				
2. Neurological disability (GNDS)	16.98	8.31	0.50 ***	-			
3. Sense of coherence (SOC-29)	130.84	29.61	−0.06	−0.35 ***	-		
4. Physical impact of MS (MSIS-29)	51.62	19.33	0.65 ***	0.66 ***	−0.22 **	-	
5. Psychological impact of MS (MSIS-29)	23.74	9.48	0.20 *	0.52 ***	−0.62 ***	0.59 ***	-

Note: * *p* < 0.050; ** *p* < 0.010; *** *p* < 0.001.

**Table 5:** Results of the Pearson’s r correlation.

	Sense of Coherence (SOC-29)				Impact of the MS (MSIS-29)	
	Comprehensibility	Manageability	Meaningfulness	Total Score of SOC-29	Physical Sphere	Psychological Sphere
Cognitive disability (GNDS)	−0.21 *	−0.28 **	−0.14	−0.24 *	0.10	0.35 **
Mood disability (GNDS)	−0.49 ***	−0.53 ***	−0.37 ***	−0.53 ***	0.24 *	0.52 ***
Visual disability (GNDS)	0.01	−0.03	−0.10	−0.04	0.12	0.11
Speech disability (GNDS)	−0.13	−0.11	−0.13	−0.14	0.10	0.22 *
Swallowing disability (GNDS)	−0.10	−0.15	−0.02	−0.10	0.05	0.20
Upper limb disability (GNDS)	0.12	0.11	0.03	0.10	0.54 ***	0.13
Lower limb disability (GNDS)	0.05	0.07	−0.16	<−0.01	0.55 ***	0.05
Bladder disability (GNDS)	−0.09	−0.09	−0.01	−0.07	0.49 ***	0.19
Bowel disability (GNDS)	−0.21	−0.15	−0.05	−0.16	0.34 **	0.22 *
Sexual disabilities (GNDS)	−0.08	−0.07	< 0.01	−0.06	0.31 **	0.12
Fatigue (GNDS)	−0.31 **	−0.27 *	−0.13	−0.28 *	0.43 ***	0.45 ***
Total score (GNDS)	−0.27 *	−0.26 *	−0.16	−0.26*	0.65 ***	0.47 ***

Note: * *p* < 0.050; ** *p* < 0.010; *** *p* < 0.001.

**Table 6:** Results of three separate stepwise regression analyses.

DV	Predictors	*Beta*	*SE*	*t* (*df*)	*p*	Model Summary		
						*F* (*df*_1_; *df*_2_)	*p*	*R* ^2^
Sense of coherence (SOC-29)	Mood disability (GNDS)	−0.43	0.08	−5.12 (132)	<0.001	22.30 (2;132)	<0.001	0.34
	Fatigue (GNDS)	−0.19	0.07	−2.59 (132)	0.011			
Physical impact of MS (MSIS-29)	Physical disability (EDSS)	0.57	0.08	7.05 (82) ^#^	<0.001	39.64 (2;82) ^#^	<0.001	0.49
	Fatigue (GNDS)	0.32	0.08	4.01 (82) ^#^	<0.001			
Psychological impact of MS (MSIS-29)	Fatigue (GNDS)	0.29	0.07	4.24 (132)	<0.001	58.44 (2;132)	<0.001	0.47
	Sense of coherence (SOC-29)	−0.53	0.06	−7.94 (132)	<0.001			

Note: ^#^ only 86 patients answered the questions about sexual disabilities.

## Data Availability

The data can be made available from the corresponding author upon reasonable request.
